# Novel Nasal *in situ* Gelling System for Treatment of Sinusitis

**Published:** 2009

**Authors:** M. R. Mehta, S. A. Surve, M. D. Menon

**Affiliations:** Bombay College of Pharmacy, Kalina, Mumbai-400 098, India

**Keywords:** Nasal *in situ* gelling systems, mucoadhesion, gamma scintigraphy, nasal residence time

## Abstract

In situ gelling systems based on temperature-dependent phase transition containing pheniramine and phenylephrine were developed using combination of Poloxamers, different cellulose polymers (HPMC) and xanthan gum. The formulations were tested for in vitro gelation, appearance, pH, drug content, in vitro drug release, mucoadhesive strength, preservative efficacy and stability. In vitro release studies revealed significant prolonged released of both drugs up to 24 h as against only 2 h with drug solution. Formulations were found to stable over period of 3 months. In vivo nasal residence time of in situ gel by gamma scintigraphy was found to be significantly higher (6 h) in comparison to drug solution (15 min). It can be concluded that Poloxamers in combination with HPMC are suitable to develop stable, safe in situ temperature-based mucoadhesive gelling systems with prolonged nasal residence time.

Almost 15% of world's population suffers from chronic sinusitis. Treatment for sinusitis is directed towards relief of symptoms; drugs used include antihistaminics, antibiotics, corticosteroids and decongestants. Several formulations for relief of sinusitis are approved for intranasal aerosol delivery by nasal spray pumps or by pMDI systems. These formulations require frequent administration due to nasal mucociliary clearance. Also, nasal decongestants may dry out the affected areas and damage tissues, and with prolonged use they often become ineffective. The tendency is to then increase the frequency of use to as often as once an hour which can cause rebound effect. The present work aimed to develop sustained release *in situ* gelling formulations of antihistaminic and decongestant combination for intranasal administration, for treatment of sinusitis, and there by overcome the frequent dosing required with conventional nasal formulations.

## MATERIALS AND METHODS

Poloxamer 407 and Poloxamer 188 (gift from BASF, Mumbai.), HPMC E15, HPMC E50 and HPMC K_4_ M (gift from Colorcon, Mumbai), phenylephrine HCl (gift from Centaur Limited, Mumbai), pheniramine maleate (gift from Supriya Chemicals, Mumbai) Mucin (Type III) from porcine stomach (Aldrich, USA), xanthan gum (C P Kelco, USA), benzalkonium chloride, glycerine, sodium chloride (S. D. Fine-Chem Ltd., Mumbai).

*In situ* gelling systems based on temperature-dependent phase transition were developed using combination of Poloxamers. In order to reduce concentrations of above polymers and to develop bioadhesive property, different cellulose polymers (HPMC) and xanthan gum were tried in various concentrations. Glycerine (humectant), sodium chloride (tonicity adjuster) and benzalkonium chloride (preservative) were also included in the formulation. The formulations were tested for *in vitro* gelation to screen the suitable polymer combinations. Selected formulations were evaluated for appearance, pH, drug content, *in vitro* drug release, mucoadhesive strength, preservative efficacy and stability. *In vitro* drug release was studied in simulated nasal secretion at 37° for upto 24 h. Mucoadhesive strength was measured by modified pan balance method with mucin (Type III) as a substrate. Preservative efficacy testing was carried out as per IP method using *E*. *coli*, *B*. *subtilis*, *C. albicans.* Selected formulations were evaluated for *in vivo* nasal irritation in female Wistar rats and nasal mucosal residence time in New Zealand white rabbits by gamma scintigraphy.

## RESULTS AND DISCUSSION

**Fig. 1:**
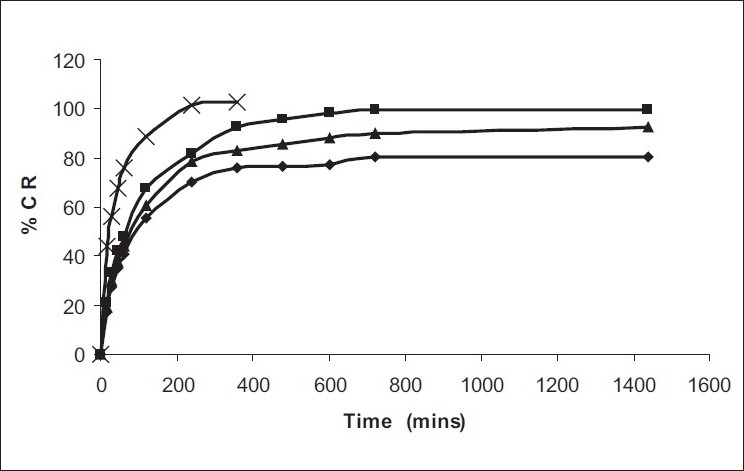
Comparative *in vitro* release profile of phenylephrine HCl Release profile of phenylephrine HCl from *in situ* gel formulation from (-◆-) PH1, (-■-) PH2, (-▲-) PH3 and solution (-x-)

**Fig. 2:**
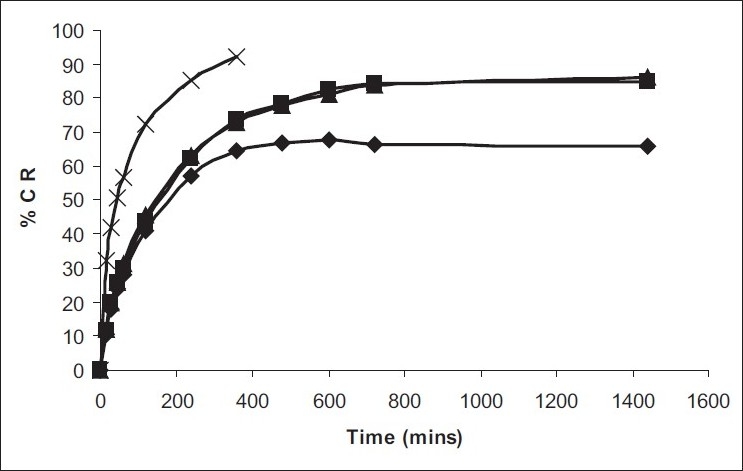
Comparative *in vitro* release profile of pheniramine maleate Release profile of phenylephrine maleate from *in situ* gel formulation from (-◆-) PH1, (-■-) PH2, (-▲-) PH3 and solution (-x-)

Among the various polymers tried, only HPMC E-15 was able to form a consistent gel at low concentration of Poloxamers (Table 1), and these three batches were selected for further characterization. pH of formulations was found to 4.8-5.0, suitable for nasal use. Mucoadhesive strength of *in situ* gel formulations was significantly improved with inclusion of HPMC. *In vitro* release studies revealed significant prolonged released of both drugs upto 24 h as against only 2 h with drug solution. Formulations were found to stable over period of 3 months (fig. 1). *In vivo* nasal residence time of *in situ* gel by gamma scintigraphy was found to be significantly higher (6 h) in comparison to drug solution (15 min) (fig. 2). It can be concluded that Poloxamers in combination with HPMC are suitable to develop stable, safe *in situ* temperature-based mucoadhesive gelling systems with prolonged nasal residence time for the treatment of sinusitis.

**TABLE 1:** *IN VITRO* GELATION TIME AND MUCOADHESIVE STRENGTH OF SELECTED BATCHES

Batch	Gelling Temperature (°)	Mucoadhesive strength (g)
PH 1	33	11.20
PH2	34	7.89
PH3	35	7.66
